# Dual-use decision making: relational and positional issues

**DOI:** 10.1007/s40592-015-0026-y

**Published:** 2015-03-03

**Authors:** Nicholas G. Evans

**Affiliations:** Department of Medical Ethics and Health Policy in the Perelman School of Medicine, University of Pennsylvania, 3401 Market Street, Suite 320, Philadelphia, PA 19104-3319 USA

**Keywords:** Dual-use research, Positional value, Relational value, Influenza, Synthetic biology

## Abstract

Debates about dual-use research often turn on the potential for scientific research to be used to benefit or harm humanity. This dual-use potential is conventionally understood as the product of the magnitude of the harms and benefits of dual-use research, multiplied by their likelihood. This account, however, neglects important social aspects of the use of science and technology. In this paper, I supplement existing conceptions of dual-use potential to account for the social context of dual-use research. This account incorporates relational and positional concerns that feature in the success or failure of dual-use. I then defend this account against foreseeable objections.

## Introduction

The dual-use dilemma in the biological sciences, in which one and the same piece of research may be used for beneficial or malevolent ends, emerged into the policymaking consciousness in 2001. Then, a group of Australian scientists studying *ectromelia variola,* or mousepox, engineered a strain of the virus that killed 100 % of immunologically naïve and vaccinated mice, and 60 % of genetically resistant mice (Jackson et al. 2001). Though the work had potential applications in controlling rodent plagues in Australia (Kerr et al. 2004; Selgelid and Weir 2010), and better understanding poxviruses—of which cowpox, monkeypox, and smallpox are all transmissible in humans—the research also had a dark side. The genetic similarity of poxviruses left open the potential for using the research to modify a human-transmissible poxvirus;^1^ a recipe for a deadly pandemic (Miller and Selgelid 2008).

Later in 2001, a series of letters laced with anthrax were mailed to Senators and media figures around the United States of America. Of the twenty-two people infected by the attacks, five died. Though an investigation into the strain used and method of delivery was inconclusive as to the identity of the attacker (Matsumoto 2003; Inglesby et al. 2002), the incident highlighted the probable ease with which bioterror attacks could be pursued (Kellman 2007).

The resulting policy discussion focused on the possibility that the life sciences were an increasingly powerful tool for would-be bioterrorists. This capacity was, moreover, deemed to be a broad problem for the life sciences. If there were particularly worrisome cases—what would later be called “dual-use research of concern” (National Science Advisory Board for Biosecurity 2007)—they were embedded in the context of a much larger technical and cultural revolution in the life sciences (National Research Council 2004).

Since then, research has appeared on the moral responsibility of scientists (Kuhlau et al. 2008), the ethics of restricting access to scientific information (Selgelid 2007; N. G. Evans 2013a), and the justifications for particular types of dual-use research (Buchanan and Kelley 2013). Yet little work in bioethics has emerged, particularly in the literature on the ethics of dual-use, that addresses the impact of the larger revolution in the life sciences on security and innovation—the province, typically, of sociology (Rappert 2007), anthropology (Rabinow and Bennett 2012), and science and technology studies (McLeish and Feakes 2008; Evans 2014).

These larger implications, however, are of significant normative and philosophical importance. The life sciences, and institutions that support and benefit from life sciences research, represent a complex network of actors jointly engaged in the project of promoting values through scientific advancement. What these values ought to be, and how we ought to achieve them, is a question of how we ought to structure our institutions—a political philosophical and ethical problem if ever there was one. Attacking this problem, however, requires a more nuanced account of dual-use potential: an account that acknowledges dual-use research in the context of joint and collective actions.

## Illustrating the problem

### The H5N1 gain-of-function studies

In 2011, two studies emerged demonstrating that highly pathogenic avian influenza H5N1 could be modified to transmit between ferrets, which are used as proxies for humans in influenza research. At present, wild H5N1 only infects humans via birds. Yet with a case-fatality rate of 59 %,^2^ the intentional or accidental release of a mammalian-transmissible version of H5N1 could be devastating. Concerns about the dual-use potential of the studies prompted the National Science Advisory Board for Biosecurity (NSABB) to initially recommend the partial censorship of the research, including the suppression of key details of the method by which the modified viruses were produced (NSABB 2011).

This recommendation generated stiff resistance from elements of the scientific community. Advocates of the 2011 studies and their successors, now known as gain-of-function research that could result in potential pandemic pathogens (GOF/PPP), have argued that the benefits of the research outweigh the risks; in 2012, this led to the NSABB recanting their decision in response to revised submissions (National Science Advisory Board for Biosecurity 2012).

Proponents claim, first, that the studies help raise awareness of the threat of the emergence of pandemic strains of disease (National Science Advisory Board for Biosecurity 2011). More significantly, GOF/PPP studies are thought, by some, to assist in the creation of therapeutic agents and vaccines that will help in the fight against emerging infectious diseases. Bill Sheridan, of BioCyst Pharmaceuticals Inc., claimed at a meeting of the NSABB in 2014 that if GOF/PPP work like the H5N1 studies were halted, pharmaceutical firms would be unable to develop antiviral drugs (Greenfieldboyce 2014). Stacey Schultz-Cherry at St. Jude’s Children’s Research Hospital made an even more ambitious claim: restricting GOF/PPP studies, according to Schultz-Cherry, could negatively impact the selection of influenza strains for seasonal vaccine production (Reardon 2014).

Yet the merits of the H5N1 studies—and similar studies carried out in 2013 (Zhang et al. 2013) and 2014 (Linster et al. 2014; Watanabe et al. 2014)—remain controversial. It is unlikely that awareness about a complex public health issue such as pandemic disease hinges on the outcome of two peer-reviewed studies. Public conversation about pandemic influenza needs to be widespread and sustained; scientists are necessary, but far from sufficient participants in this effort (Evans 2010; Evans 2014).

Second, critics question the ability of GOF/PPP studies to facilitate prediction of the emergence of pandemic (much less seasonal) strains of infectious disease. They contend that there is little guarantee that a pandemic virus will emerge with genetic markers mirroring the changes produced in GOF/PPP studies (Evans 2013a). Moreover, genetic changes found in the 2012 studies have been shown to produce differing, at times opposing, phenotypes in influenza in other studies. Epistatic interactions—in which genes interact with their genetic background to produce particular behaviors—generate indeterminate results in the case of GOF/PPP studies (Lipsitch and Galvani 2014). This undermines the value of the studies vis-à-vis surveillance efforts, or vaccination and therapeutics development.

Finally, the purported benefits of dual-use research are typically best understood as complex joint actions that promote human values. In the case of GOF/PPP studies, a lack of *institutional capacity* in global public health infrastructure frustrates surveillance and vaccination efforts, which in turn frustrate attempts—setting aside the above technical issues—to convert dual-use GOF/PPP research into productive and beneficial outcomes. Vaccines are difficult to produce in quantities that would allow for adequate response in a disease pandemic, much less distribute (Fedson and Dunnill 2007). Industry, regulators, academia, and governments either lack the vision or strength of cooperation to pursue new vaccine development strategies (Osterholm et al. 2013). Disease surveillance requires a health system that is able to reliably—and ethically—collect and collate patient data, and citizens that are informed and empowered to seek health care facilities in a timely fashion (Khan 2011).

An important reason not to engage in the post hoc censorship of dual-use GOF/PPP research is that the World Health Organization’s Pandemic Influenza Preparedness framework is predicated on information sharing between countries (World Health Organization 2012). This still leaves open the question, however, of the benefits the studies provide in terms of public health, relative to any attendant risks of misuse. Assessing this dual-use potential, however, is not straightforward. How should we account for the range of factors that inform particular uses of dual-use research?

### Synthetic biology

Dual-use potential can manifest in communities as readily as individual pieces of published research. As biotechnology becomes ever more ubiquitous and personal, an increasing number of domestic institutions are likely to come into contact with the field, and thus with dual-use research. Nowhere is this more apparent than in the development of synthetic biology: the design and construction of new biological parts, devices, and systems, as well as the redesign of existing, natural biological systems (Way et al. 2014).

Advances in synthetic biology are increasing access to, and participation in, the life sciences and biotechnology. The International Genetically Engineered Machine (iGEM) competition, due to enter its twelfth year in 2015, involves teams of young scientists fielding competing designs of biological devices built from a toolkit of standard biological parts (Carlson 2010). Other movements, such as the “Do-It-Yourself” and “Garage” biology movements seek to encourage biological experimentation and innovation by nonprofessionals, for fun or profit.^3^

Synthetic biology brings with it, however, significant dual-use potential. An extreme possibility is that synthetic biologist could one day synthetize smallpox, either the wild-type or a genetically augmented strain, such as one that makes use of the discoveries of the 2001 mouse pox virus (Samuel et al. 2009). Less catastrophic, but still concerning—and probably more likely—would be an epidemic of lesser harms: a rash of hospital admissions from individuals accidentally infecting themselves or others with pathogens, or crimes of passion involving difficult to trace but easily obtainable biotechnology.

In case this last scenario seems far-fetched, consider a story of chemistry. In the first half of 2014, three individuals in the US were arrested for distilling lethal doses of the ricin toxin from castor beans. One case was a curious student at Georgetown University, who, though suffering from psychological distress, allegedly harbored no malevolent intentions (Jouvenal and Marimow 2014). The other two, however, involved a plot to kill a romantic rival (Chang 2014) and an attempt to kill a woman and her unborn child (Passoth 2014). Fortunately, no one was harmed. If this is what can be done with basic chemistry today, however, we ought to be concerned about future uses of biotechnology.

Accessible synthetic biology challenges traditional paradigms of security. It is unlikely that regulatory devices such as professional codes, export controls, or classification will be effective in the context of a deskilled, deprofessionalized community of practitioners. Other solutions will be needed: preventative options such as high-school bioethics and biosafety education, and more responsive disease surveillance; palliative measures like the development of new forensics, and hospitals better equipped for biological containment. The institutions where these options manifest, however—schools, clinics, police stations, and hospitals—have not been examined in terms of the ethics and logistics of dual-use regulation, much less funded or equipped for such an undertaking.

Dual-use potential *in* synthetic biology—that is, the dual-use potential of particular experiments inside the field *of* synthetic biology—is rapidly becoming the dual-use potential of synthetic biology. Certain broad elements of synthetic biology, driven by the aim to create a predictable engineering discipline out of the life sciences, have the capacity to deskill the life sciences in a way that enables malevolent actors. This dual-use potential is not one of individual experiments, but latent in the structure of synthetic biological communities (Evans and Selgelid 2014).

To date, the bioethics literature on dual-use has not accounted for issues specific to dual-use as a community-level concern. Mention is made, however, of a need to “design ethics in[to]” our social institutions to prevent or mitigate the harms associated with the misuse of emerging technologies (Van den Hoven 1997; Miller and Selgelid 2008). Doing so, however, requires understanding the role these institutions play in dual-use. This, in turn, requires a better account of dual-use potential.

## Conceiving of dual-use potential

Though the literature lacks consistent terminology on what constitutes “dual-use potential,” a common thread is that dual-use potential refers to the likelihood for scientific research to be used in beneficial or harmful ways. This serves as a starting point for my discussion; below, I outline some features of dual-use potential that are common in the current debate.

To begin, Miller and Selgelid (2008, pp. 34–35) note that:

The danger attendant upon a given dual-use research program can be crudely quantified by determining the probability, be it low, medium, or high, of a given untoward outcome, and multiplying this probability by the (quantified) disvalue (or disutility) of that outcome.

Here, dual-use potential is conceived of in explicitly consequentialist terms: as a product of the likelihood a set of states of affairs attaining, and the magnitude of the value those states of affairs possess (Pettit and Brennan 1986). Though accounts from a (primarily) nonconsequentialist ethic do exist in the debate (e.g., Ehni 2008), a focus on *use* places a strong emphasis on the moral weight of consequences.

Resnik (2013, p. 26) notes that this conceptualization is common, particularly in relation to restricting access to scientific publications:

A common way of thinking about restrictions on scientific publication is to assess the potential benefits and risks of different choices and then to select the option that has the greatest balance of benefits over risks…

…Risk is usually defined as the product of the probability and degree (or severity) of harm.

Resnik’s account, however, gives additional insight into dual-use potential as an aggregate of both beneficial and the harmful uses. Good policies will change the dual-use potential of research by mitigating risks, while promoting—or, if that isn’t possible, minimally interfering with—beneficial uses.

Tucker (2012, pp. 67–69) outlines four parameters for identifying risk of misuse, as part of a decision framework for regulating dual-use research. These are (1) accessibility (of the dual-use research or technology), (2) ease of misuse, (3) magnitude of potential harm, and (4) imminence of misuse. Users will differ in terms of their ability to access and utilize science and technology, and their capacity to achieve significant outcomes through that use.

Dual-use potential, then, can be characterized as:

1. Forward-looking and consequentialist, a function of;
  - The likelihood of a particular use;
  - The magnitude of the value of that use;
2. An aggregate of both the beneficial and harmful uses of dual-use research;
3. Existing in the context of a set of users with different capacities to bring about certain outcomes.

These features are often tacit in discussions about dual-use, but they describe elements of dual-use potential that are important to decision-making.

## A deeper account

It is not clear, however, how to account for varied layers of implementation between dual-use research and end-user. Nor is it clear how we account for where, or how, we are justified in interfering with or regulating the pursuit of novel, rapidly evolving life sciences research and development. Consequentialism provides one answer: the likelihood that scientific knowledge will promote some ultimate value is a function of the path science takes to percolate from conception to implementation: given sufficient information about each conditional probability in the sequence, we can calculate the expected value (as a product of both likelihood and magnitude of value) of a particular use. We can do this for all potential paths (successful or otherwise) the research might take, and determine the expected value.

In the presence of complexity and uncertainty, however, computational biases can quickly frustrate this method.^4^ For example, actors are likely to over-weight the value of goods that are proximate and personally meaningful to them—such as a scientist’s own enjoyment of the scientific process—over those that are temporally displaced and effect strangers (Babcock et al. 1995). Leaving ethical decision making about dual-use potential to scientists—or anyone other single group—then, is likely to skew our decision making process (Kitcher 2001).

Implementation, moreover, is not a stochastic process. Rather, actors and institutions play *roles* in realizing or frustrating the uses of dual-use research. We should be able to provide information about these roles, and their moral significance. Doing this requires supplementing our framework with an account of those systems in which dual-use research is embedded.

### Relational properties

Certain uses of dual-use research are contingent on sets of actions that, taken together, create valuable outcomes from research. Not all uses are joint actions—the hypothetical mad scientist, seeking to unleash a plague against the target of their hateful beliefs, may act independently of others. Many uses, however, rely on joint or collective actions.

The philosophical literature on joint actions is vast, and I will only introduce these terms as illustration of the varied nature of joint actions as they apply to the current debate. Some joint actions are *collective* actions, between agents that are both mutually responsive and committed to a joint end (Bratman 1991). Communities of scientists may be considered to be engaged in a collective action, insofar as they build on each other’s work in the name of advancing the shared project of science (Miller 2013; Kitcher 1990).^5^ Even when scientists compete to be the first to a discovery, they will do so within the framework of this shared project (Mitroff 1974).

Other joint actions are *shared cooperative activities*, where, in addition to the requirements for collective action, agents are mutually supporting in completing their ends. In some cases, such as collaborative research teams, scientists fit this description. In most cases, however, the relationship between researchers and others that apply research to productive, nonscientific outcomes is weaker.

To understand the importance of joint actions to dual-use potential, let’s return to the prospect of GOF/PPP research leading to better vaccines—and thus better public health outcomes. Moving from scientific research to “vaccination” is a collective action that intentionally promotes the health of a population against a disease. To achieve this end, scientists conduct continuing research on a pathogen, periodically publishing their results. These publications may be integrated into the research and development of novel vaccines, which are tested—multiple times—for efficacy and safety. Vaccines are manufactured, distributed, purchased, and stored; doctors, nurse practitioners, and pharmacists administer those vaccines. Those who administer vaccines may undertake education on the vaccine’s effectiveness, mechanism of action, longevity, and adverse effects. Public health authorities conduct vaccination campaigns using a range of strategies from the merely persuasive through to the coercive. Epidemiologists monitor the uptake of vaccines, the spread of the targeted disease, prevalence of adverse effects, and associated costs of the vaccination program. Regulators initiate post-market surveillance of vaccines.

Bioethicists, of course, debate the ethics of most of the above!

All of these actors work towards the ultimate goal of vaccination. They do so, moreover, collectively. That is, they are mutually responsive, and committed to a shared end. It is unlikely that they are committed to a shared cooperative activity, as their ability or desire to be mutually supportive comes in greater or lesser degrees. But insofar as these actors are intent on vaccines and vaccinations occurring, and playing their part in that process, they are engaged in a collective action.

Many of these roles are necessary for completing the collective action of vaccinations. Some of these roles, moreover, are necessary to *promote* particular values in the context of the final outcome. Others, such as the actions of pharmaceutical regulators, protect against other undesirable outcomes such as adverse reactions or premature loss of vaccine effectiveness; they protect the good of human health against future loss. Security—the protection of some intrinsically valuable good against loss (Selgelid 2012)—is the ultimate purpose of developing and using vaccines: we want to protect our health against future loss. Part of this security includes securing against possible harms caused by vaccinations. Even if a vaccine provides great benefit at little cost, it is arguably deficient *qua* vaccine unless it is subject to a particular level of testing, because we fail to promote security of our present and future health in the above sense.

Dual-use potential that depends on joint actions, then, features a *relational* component between dual-use research and the joint mechanisms that produce a given use. In the case of GOF/PPP studies—at least as far as an appeal to the value of these studies relies on their use in pharmaceutical or vaccine production—a significant concern arises about the likelihood that vaccines will be distributed to (developing) countries where pandemic disease strains are most likely to arise. There is also concern about the degree to which current vaccine manufacturing technology could produce sufficient quantities of a vaccine to combat a pandemic. In either case, the failure of one element of the joint action jeopardizes the ultimate ends of vaccines. It is not meaningful to talk about the application of the H5N1 studies in terms of vaccines, unless we account for these other factors.

Importantly, these joint actions need not be cooperatively loaded (Bratman 1991). That is, joint and collective actions do not require some intention to act together cooperatively. This is particularly important in terms of complex public health processes, in which role holders may span institutions. Insofar as scientific researchers, businesspeople, healthcare workers, and government officials occupy different institutional roles, their intentions may differ even if they are all working to produce the same end.

It is clear that some paradigm cases of dual-use—and, in particular, those that have caused most concern—involve in collective action. In the case of the mousepox study, the lead researcher was involved with Australian government efforts to enhance the *myxoma* virus’ capacity to control rabbit populations (Selgelid and Weir 2010). The H5N1 studies were both funded by the National Institute of Allergy and Infectious Diseases, and the purported public health benefits of the studies were described from the outset (Herfst et al. 2012; Imai et al. 2012). In both cases, the research was performed in the context of government-funded research with specific ecological or public health aims. The researchers may also intend to advance their careers, and create interesting scientific knowledge that satisfies the epistemic norms of their community; insofar as the creation of vaccines is an important, *justificatory* end, however, their role in this collective process is significant to the project of understanding dual-use.

Joint actions, further, need not have beneficial ends. A state weapons program is an immense joint action that may involve dozens of laboratories, munitions sites, military installations, intelligence units, and support facilities. Biopreparat, the clandestine Soviet biological weapons program, comprised numerous civilian and military laboratories, production facilities, scientific institutions, bureaucratic organizations, as well as the Soviet intelligence community (Leitenberg et al. 2012). Accounting for the malevolent uses of dual-use research requires accounting for these joint actions, where they might occur.

When we consider the misuse of biological research by bioterrorists, however, the relation between science and terrorist attacks is less likely to be one of collective action. Rather, malevolent actors are arguably more likely to be *parasitic* on life sciences research—that is, terrorists appropriate new science and technology, rather than acting in concert with scientists. In this case, we want to sever the weak relational properties that connect the (beneficent) life sciences with aspiring bioterrorists. This implies a different, adversarial relationship between actors; the good we wish to promote depends on the position of one agent over another.

### Positional concerns

Relational concerns provide an account for the way that dual-use incorporates joint action. When we consider the security implications of dual-use research, however, we engage in a process of attempting to *stop* an individual or joint action such as bioterrorism. This is not in itself a joint action, as though we are mutually responsive, we are not committed to a same joint end.^6^ Rather, the opposite holds: the value of my stated end in part depends on ensuring that my end succeeds *and* those of others—aspiring bioterrorists—fail. Our (valuable) health—and its security— is *positional,* that is, it’s position is dependent on someone else lacking something (here, the capacity to cause mass harm) (Fiorito and Vatiero 2013).

The simplest cases of positional concerns in dual-use research and technologies emerge in the context of an older sense of “dual-use”: one that distinguishes between military and civilian uses of a technology (Molas-Gallart 1998). Here, the value of dual-use technologies is derived in part by denying others possession of that technology (See e.g., Knowles 2012). In other cases, institutions monitor or regulate particular uses, through criminal sanction or force, such as law enforcement agencies monitoring and arresting potential bioterrorists.

Other, less intuitive examples of positional factors in promoting value exist. For example, codes of conduct are intended to promote the ethical pursuit of biotechnology. While these may be considered in one sense in relation to the joint end of pursuing (good) science, they also have a positional element. That is, when good conduct by laboratory members is promoted, it assists in deterring and policing the malevolent or reckless ends of others (Lentzos 2008). Codes are, arguably, partly positional because they frustrate harmful uses, in addition to promoting beneficial uses.

As an example of positional concerns that impact dual-use in a broad sense, consider education. Synthetic biology communities continue to conduct outreach with school-age practitioners; we should expect this trend to continue as biotechnologies—like information technologies—become ubiquitous. Schoolteachers, in this sense, are partly responsible for educating future users of dual-use technologies—and in affecting how they conduct themselves in the lab.^7^ Introducing biosafety, or bioethics, education into schools presents on option in promoting security by generating an awareness of biosafety and security issues early, in the same way that biosecurity can be enhanced inside professional communities through social forces.^8^

Importantly, pharmaceutical regulation is not *merely* positional, under my account, to the attainment of uses such as vaccination. As before, vaccines—as therapeutic agents—are required to have a certain standard of safety and efficacy in order to be considered worthwhile as vaccines. A well-functioning regulator provides that security, which is part of what it is to be a worthwhile use of this science and technology. The sense in which regulation is positional is in regulating the distribution of therapeutic products, (ideally) weeding out those that are genuinely beneficial from those that are snake-oil or otherwise misleading in their claims for a person’s health.

### Common properties

Relational and positional concerns share some common features.

First, relational and positional properties of dual-use potential are matters of degree. Dual-use potential can be more or less dependent on its relation to joint actions, and those joint actions can be of greater or lesser complexity. Dual-use research can also be secured in a desirable fashion to a greater or lesser degree. Moreover, institutions or institutional arrangements may be more or less positional in terms of their ability to frustrate the ends of a particular use.

Next, relational and positional properties apply to given uses, or *sets of uses.* This means that, in part, dual-use potential can be defined in terms of a set of uses, rather than a particular instance of research. We can talk about dual-use research, but also dual-use technologies, communities, fields, research programs, methodologies, and so on. Imputing dual-use potential with relational and positional dimensions allows us to better account for communities such as synthetic biology, which generate concerns about the misuse of science and technology in a more systemic sense than, say, the H5N1 studies (Tucker 2011).

The ethical considerations latent in dual-use research, moreover, may differ from those posed by dual-use technologies, communities, and so on. For example, researchers within a university setting will be protected under their rights to academic freedom (Miller 2010, p. 225); biotechnology companies are not subject to the same type of protections. A more nuanced account of dual-use allows us to pick out moral properties specific to particular kinds of scientific and technological pursuit, as well as draw connections between seemingly disparate endeavors that are bound together by common sets of uses.

Relational and positional properties of dual-use are contextual. That is, relational properties of the clandestine bioweapons program—say, the infrastructure that keeps the program secret—may, in turn, be positional to the security aims of a rival state. Likewise, the success of public health agencies in communicating the merits of vaccination may be considered to be adverse to aspiring bioterrorists. Whether something is relational, positional, or both is dependent on the locus of our decision making process.

Rather than being viewed as turning dual-use into an untidy mess, or a slide into moral relativism, however, we should look at positional and relational features in terms of the values they promote. In this regard, relational and positional features of dual-use are compatible with existing concerns. There is nothing about these properties that tells us whether or not a specific property will be morally justified, in the same way that claiming something has “instrumental value” gives us no knowledge about whether or not the end to which something is instrumental is good or bad. Positional and relational accounts of value must, in turn, be wedded to a robust anatomy of value. While I have defended a plural system of values elsewhere (Evans 2013b), these relational and positional properties do not depend on those values for my argument to hold here.

### Fleshing out the account: institutional capacity

Focusing exclusively on direct regulation of dual-use research and researchers gives us limited insight into the possibilities for preventing the misuse of science and technology. Moreover, the uses of dual-use research—about which we are primarily concerned—depend on the interplay between joint (and cooperative), and adversarial agents.

What this means for an analysis of dual-use is that institutions such as regulatory authorities, criminal justice and legal systems, and scientific research funding, should be considered in wide accounts of the ethics of dual-use research and technology. We should also consider how the internal structure of different research communities—corporate, academic, medical, military, government, and emerging deprofessionalized movements—promotes the creation, dissemination and use of dual-use research and technology.

Institutional capacity provides a better understanding of how we promote the beneficial uses of dual-use research over the harmful. Rather than a mere trade-off between security and science (e.g., Resnik 2013) we should conceive of dual-use in terms of broader plural and conflicting ends. In this context, the potential trade-off between security and science at the point dual-use research is scheduled to be published is only one class of decision we could make. Other options may include: (a) designing security into the institution of science; (b) empowering some other institution to moderate the interaction between scientific and security concerns; (c) redesigning the system of funding of scientific research to promote security; or (d) empowering the palliative arm of security, such as forensics and surveillance.

The degree to which we mobilize other institutions to address dual-use issues is, itself, an ethical issue. For example, the attempted synthesis of public health and security systems has generated concerns about the “securitization of public health,” understood as the theory and practice of public health being considered in security terms (Fidler and Gostin 2008, p. 122). Among other concerns is the possibility that securitizing health could derail, or otherwise corrupt, the pursuit of the legitimate goals of health care. One could make similar arguments about incorporating the rubric of security into other social institutions, such as education or a free press.

Importantly, promoting security is not synonymous with occupying a security *role*: the concerned citizens that reported the ricin manufacturers certainly promoted security, even if they did not function in a security role. In identifying the degree, and way that institutions could play a role in depriving malevolent actors of recruits or materials, we have to take particular care in how we conceive of this role, relative to the existing—justified—ends of our social institutions. While securitization is a legitimate concern, not all institutional frameworks that promote security are, by necessity, security-specific institutions (and their role-holders).

A focus on the roles of institutions dovetails into another potential concern about my account—that I have moved discussion of the ethics of dual-use in a way that eschews the value of science for its own sake. Science is inherently valuable, in that people enjoy doing science for its own sake. It is also intrinsically valuable—the world is surely better with science in it, simply for its being there. The knowledge that science provides us is also valuable for its own sake, and as it contributes to our wellbeing. That I’ve evaluated science solely in terms of its applications threatens a kind of expediency, framing science as a mere piece in an institutional puzzle. If that were the case, then science could be censored or restricted because of security concerns, when what is creating the problem is a deficiency in some other institutions.

Expediency says little of ethics, however; this, after all, is an ethical project. That the application of scientific research may be frustrated by other relational or positional factors *is* important, but it is surely not the only thing that is important. Our decision-making will still need to incorporate the values that are promoted by our actions over a range of possible strategies for preventing the misuse of science and technology. There is no in principle reason why scientific research will always be the locus of our regulation. My account, here, is intended to supplement existing decision making processes; it is not intended to replace existing moral concerns such as academic freedom and freedom of inquiry, freedom of speech, and scientific autonomy (Resnik 2008; Miller and Selgelid 2008) that provide a presumptive case for protecting scientific research.

Moreover, with the exception of the potential intrinsic value of scientific knowledge, valuing knowledge relies on, and is itself a product of, social institutions. Classrooms, public outreach, and high-quality scientific journalism all serve to educate us and proliferate scientific knowledge; this, in turn, promotes the value of science for its own sake. Science itself is the outcome of a society-wide joint action of engaging and training future scientists, equipping them with the right materials, and promoting their work. Treating it as such gives greater credence to the idea that science is valuable for its own sake, and provides a better account for the value of science in terms of its impact on other worthwhile projects.

Insofar as there is the capacity for an institution to causally effect dual-use potential, and those institutions play important roles in promoting values, there will be a series of ethical questions as to how we accomplish our goals in a morally justified manner. While the best explored of these interfaces to date is between national security and health, the interfaces between scientific research, public health, law enforcement, media, education, and so on all require exploration.

### Moving forward

Dual-use potential depends on the value of the uses of scientific research. These uses, however, often occur within institutional frameworks. Our ethical discourse and policymaking about dual-use should account for those institutions, and their capacity to enable or frustrate the use—and misuse—of scientific research.

In moving forward with dual-use and its regulations, practitioners should start to focus on the supporting infrastructure that enables the use of scientific knowledge. Much of this is necessarily empirical and interdisciplinary: the social, organizational, and economic mobilization of science to promote human values. In this paper, I’ve provided a rough sketch of these relational and positional considerations with an eye to application in specific cases. The next step is to detail how specific considerations impact on institutional behavior and ethical practice in science.

## Footnotes

1 This was accomplished in the United States with cowpox, though the research was never published. See Connell (2012, pp. 191–192).

2 This rate is contested. However, recent work has indicated that the 59 % rate is—if not definitive—a good estimate of the fatality rate. (See e.g., Khuntirat et al. 2011; Nasreen et al. 2013).

3 Of these groups, DIYBio.org is one of the more developed; the site includes its own biosafety forums. For garage biology, see Carlson (2010), and Ledford (2010).

4 These issues have been discussed by Douglas (2013) and Smithson (2013) separately. Using expected value treatments, or modeling dual-use through game theoretic models (respectively) may still be valuable—but there are important limits on these methods as a means of ethical decision-making.

5 Of course, there are many ways that scientific research can emerge. I also don’t discount the presence of fraudsters and charlatans in science. But that doesn’t change the moral framework; in fact, that’s a reason *why* fraudsters are bad: they divert or corrupt the institution of science.

6 This contrasts with adversaries committed to a mutually agreed-upon end. Bratman (1991) notes that, for example, playing chess is an example of a collective action. Even though you and I are adversaries, we are committed to the same end: the resolution of a game according to a mutually agreed-upon set of rules.

7 They aren’t the only ones responsible, of course, but in this example I’ll limit my account to teachers.

8 This, as a matter of strategy (although not necessarily ethics), should come as no surprise. In the opening of *The Art of War*, Sun Tzu notes the first factor that governs the outcome of war is Moral Law governing the community. Community building, in a very real sense, is central to strategies promoting positional values.
